# Adverse events administering glucagon-like peptide-1 receptor agonists: a cross-sectional study

**DOI:** 10.1093/haschl/qxag023

**Published:** 2026-02-03

**Authors:** T Joseph Mattingly, Emeka Elvis Duru, Rena M Conti

**Affiliations:** Department of Pharmacotherapy, University of Utah College of Pharmacy, Salt Lake City, UT 84112, United States; Department of Pharmacotherapy, University of Utah College of Pharmacy, Salt Lake City, UT 84112, United States; Questrom School of Business, Boston University, Boston, MA 02215, United States

**Keywords:** GLP-1 receptor agonists, biopharmaceuticals, adverse events, patient education, pharmacy, compounding

## Abstract

**Introduction:**

Rapid increased utilization of GLP-1s by US patients has raised safety concerns, in addition to challenges related to supply shortfalls starting in March 2022.

**Methods:**

We analyzed publicly available FDA Adverse Event Reporting System (FAERS) data from January 2015 through December 2024 to describe adverse events where GLP-1s were the primary suspect and compared them with events involving injectable insulin products.

**Results:**

Among the 112 532 reports analyzed, GLP-1s were associated with a higher share of administration-related reactions (63%) compared to insulin (39%). Reports of dosing issues and administration errors increased for GLP-1s beginning in Q4 2022 and rose further in 2023 and 2024, patterns not seen for insulin. Increases coincided temporally with the period of national GLP-1 shortages. Increases in reporting volume may reflect increased utilization rather than increased risk as FAERS lacks exposure denominators.

**Conclusion:**

The shift toward administration-related and dosing-related reports underscores the importance of patient and provider education and continued regulatory attention to the use of these drugs even as supply shortfalls resolve. Ongoing post-marketing surveillance remains essential to monitor safety signals.

## Background

The launch and increasing use of glucagon-like peptide-1 (GLP-1) receptor agonists for the management of type 2 diabetes and, more recently, for weight loss has generated widespread attention in clinical practice. These drugs have been associated with substantial improvements in glycemic control and weight reduction. Increased popularity of these drugs have garnered more attention on social media, celebrity influence, and more people seeking these products outside of insurance coverage and reimbursement and for off-label use.^1-3^ Patients are also seeking and obtaining these drugs outside of traditional medical channels, including health spas and online vendors selling compounded products, typically at lower prices.^4^ Some of these vendors may be operating illegally or selling adulterated products.^5,6^ The IQVIA Institute's 2024 Use of Medicines in the US report estimated that nearly 100 million prescriptions were dispensed through illegal online pharmacies in 2023, almost 50% more than in 2019, underscoring the growing scale of unregulated access and the associated risk of counterfeit or adulterated products.^7^

In 2022, several GLP-1s, including the US Food and Drug Administration (FDA) approved semaglutide products Wegovy^®^ and Ozempic^®^, were placed on the FDA drug shortage list.^8^ Wegovy was added in March 2022 and Ozempic followed in August 2022.^9^ Supply challenges have contributed to patients seeking alternatives from compounding pharmacies. With increased use, safety concerns have emerged regarding GLP-1s, including compounded versions and particularly related to dosing errors. FDA issued an alert highlighting cases where patients inadvertently administered higher doses than prescribed due to mistakes in drawing medication from multidose vials.^10^ While this risk is not unique to compounded GLP-1s, the increasing availability of these medications from nontraditional sources may increase the likelihood of such errors due to inadequate patient education among other concerns.

In this study, we analyze publicly available national post-marketing surveillance data from the FDA Adverse Event Reporting System (FAERS) to characterize the levels and trends of real-world adverse event reports associated with GLP-1s. FAERS is a national post-marketing pharmacovigilance database used to support detection of potential safety signals after drugs enter routine clinical use. It includes adverse event reports voluntarily submitted by health care professionals and consumers, and manufacturer-submitted reports, and uses standardized coding to classify reported reactions and medication-use problems. Importantly, FAERS does not include population-level exposure denominators (eg, the number of users, prescriptions, or person-time), and the FDA does not recommend using FAERS to estimate adverse event incidence or comparative risk.^11^ As a passive reporting system, FAERS is subject to underreporting, variable completeness, differential reporting across products and event types, and temporal changes in reporting behavior—limitations that can influence absolute report counts.^12^ Nevertheless, FAERS is widely used for hypothesis generation and for characterizing patterns in reported events over time, particularly when analyses focus on changes in the composition of reports rather than on incidence.^12,13^

We focus on patterns of safety signals related to the administration, dosing, or product dispensed and compare reports events to injectable insulins. Insulin is an imperfect but informative comparison group for this analysis. Unlike GLP-1 receptor agonists, insulin therapy often involves complex dose titration and carries a well-recognized risk of hypoglycemia with dosing errors. Importantly, however, insulin administration practices, delivery devices, and patient education requirements have remained relatively stable over the study period and the patient population using insulins and GLP-1s may be overlapped given their clinical us treating diabetes. As a result, insulin serves not as a benchmark for dosing complexity, but as a contemporaneous comparator for changes in adverse event reporting related to the administration of injectable therapies in plausibly overlapped populations. If the observed shifts in administration- and dosing-related reports among GLP-1s reflected broader changes in reporting behavior, pharmacovigilance practices, or self-injection norms, similar changes would be expected among insulin reports. The absence of such shifts in insulin reporting patterns strengthens the interpretation that the changes observed for GLP-1s reflect class-specific dynamics rather than system-wide reporting artifacts. Prior FAERS-based analyses have successfully been used to monitor drug shortages and associated safety events, including studies of heparin supply disruptions and adverse outcomes.^14^

## Study data and methods

In this cross-sectional study, we used January 2015-December 2024 FAERS quarterly extract files to identify all adverse events reported to the FDA that included a GLP-1 or insulin product as the primary suspect in the report.^15^ The analysis utilized publicly available post-marketing surveillance data from FAERS, which captures adverse events reported by health care professionals, consumers, and manufacturers. As a voluntary reporting system, FAERS is valuable for detecting safety signals and monitoring temporal trends in adverse event reporting but cannot measure true event incidence or rates.^15^ The study population consisted of all FAERS reports filed during the study period that identified GLP-1s as the primary suspect in adverse events. We also identified adverse event reports related to insulins. Reports that did not specify GLPs or insulin agents as primary suspects were excluded (eFigure S1). Our analytic sample focused on GLP-1 and insulin monotherapy reports. Consequently, we excluded FAERS reports involving combination products (eg, semaglutide/cyanocobalamin or semaglutide + vitamin B12) to ensure sample consistency over time and mitigate attribution ambiguity.

For the included adverse events, we extracted variables from the FAERS database, including reporters’ gender, drug information, reaction type, primary indication, and report date. GLP-1 and insulin products were identified using the primary suspect drug field and matched by generic and brand names. All products included in the analysis are listed in eTable S1 in the Supplementary material.

Primary reaction for each event was extracted from the Reaction Files for each quarter. At the event-level, reaction data describes adverse events using the Medical Dictionary for Regulatory Activities (MedDRA). FAERS adverse event reports may include multiple reported reactions per case.

For this analysis, we were interested in specifically evaluating adverse events related to: 1) Administration-Related Reactions; 2) Dose or Prescribing Issues; or 3) Product-Related Issues. We define “administration-related reactions” as reactions directly associated with the method of drug delivery, including problems with injection technique, reactions at the injection site, device handling issues, and complications from incorrect administration timing or procedures. Also included are complications arising from incorrect administration timing or procedures followed by healthcare professionals or patients. We define “dose or prescribing issues” as adverse events stemming from prescribing errors or dose management errors. It includes overdosing, underdosing, or dosing at incorrect times, which can result from prescription errors, pharmacy dispensing mistakes, or patient misunderstanding of dosing instructions. Finally, we define “product-related issues” as adverse events that are related to the pharmaceutical formulation of the drug, including issues due to drug stability, expiration, storage conditions, or suspected quality defects. These categories were selected a priori to capture medication-use and product-related problems that may arise from deviations in administration, dosing, or handling practices, rather than expected pharmacologic adverse effects of GLP-1 receptor agonists. A complete list of all MedDRA terms included in this category is provided in eTable S2 in the Supplementary material.

Reports without any MedDRA term mapping to these categories were excluded from the analytic sample. Sample inclusion criteria was intentional: our objective was not to characterize the overall clinical adverse effect profile of GLP-1 receptor agonists (eg, gastrointestinal symptoms such as nausea and vomiting), but rather to focus on medication-use problems and product-quality concerns that are plausibly influenced by changes in access, dispensing, packaging, and patient education during the shortage period. Accordingly, the findings should be interpreted as describing trends in this targeted subset of reports rather than the full spectrum of GLP-1–related adverse events.

Descriptive statistics were used to summarize the data. Adverse events were assessed as a proportion of total reports involving GLP-1 receptor agonists and insulin. Chi-square tests were used for categorical variables. Specifically, we compared gender, primary indication (diabetes, weight loss, or unknown indication), and primary reaction group (defined above) for both the GLP-1 group and Insulin group. All statistical tests were two-sided, and a *P*-value of less than 0.05 was considered statistically significant. To complement our statistical analysis, we visualized reporting trends from January 2015 through December 2024 using yearly intervals. Stacked bar graphs were generated to display the distribution of reaction categories within each year, enabling an observational assessment of temporal patterns without formal statistical testing. These visualizations provide a clear depiction of changes in adverse event reporting for GLP-1 receptor agonists and insulins, highlighting periods of increased reporting and shifts in reaction types. All figures were created in R using the ggplot2 package. This draft is aligned with the recommendations of the Strengthening the Reporting of Observational Studies in Epidemiology (STROBE) statement for reporting observational studies, focusing on clarity and transparency in the description of the study design, setting, participants, variables, and methods employed.^16^

## Study results

From January 2015 to December 2024, we identified a total of 112 532 adverse events that met our inclusion criteria, with 70 955 (63.1%) GLP-1 suspected product events and 41 577 (36.9%) insulin suspected product events. A higher prevalence of females was noted in the GLP-1 group compared to the insulin group (65.7% vs 57.4%, *P* < 0.0001) along with a higher prevalence of indications labeled as “unknown” in the reports (Table 1). The distribution of primary reaction groups was significantly different between the GLP-1 and insulin groups. Administration-related reactions were more prevalent in the GLP-1 group (63.0% vs 38.7%, *P* < 0.0001), while dose or prescribing issues (21.1% vs 29.9%) and product-related issues (16.0% vs 31.7% respectively) were more frequently reported with insulins compared to GLP-1s.

**Table 1.** Comparison of GLP-1 and Insulin event reports related to product, dosing, or administration.

	GLP-1 Group *n* = 70 955 Count (%)	Insulin Group *n* = 41 577 Count (%)	*P*-value
**Female (Yes)**	43 911 (65.7)	21 737(57.4)	<0.0001
**Primary indication**			
Diabetes	29 407 (41.4)	26 987 (64.9)	<0.0001
Weight loss	3374 (4.8)	0 (0.0)	
Other or unknown indication	38 174 (53.8)	14 590 (5.1)	
**Primary reaction group**			
Administration-related reactions	44 676 (63.0)	16 084 (38.7)	<0.0001
Dose or prescribing issues	14 960 (21.1)	12 308 (29.6)	
Product-related issues	11 319 (16.0)	13 185 (31.7)	
**Reporter type**			
Consumer	62 612 (88.2)	35 957 (86.5)	<0.0001
Health care professional	3690 (5.2)	3708 (8.9)	
Other or unknown	4653 (6.6)	1912 (4.6)	
**Reports by year**			NA
2015	1997 (2.8)	5941 (14.3)	
2016	2000 (2.8)	1767 (4.3)	
2017	3310 (4.7)	2252 (5.4)	
2018	4296 (6.1)	4529 (10.9)	
2019	4760 (6.7)	4068 (9.8)	
2020	5840 (8.2)	3883 (9.3)	
2021	6161 (8.7)	4763 (11.5)	
2022	8409 (11.9)	5219 (12.6)	
2023	15 313 (21.6)	4949 (11.9)	
2024	18 866 (26.6)	4202 (10.1)	
**Product active ingredient**			NA
Tirzepatide	28 349 (40.0)	NA	
Dulaglutide	22 859 (32.2)	NA	
Exenatide	15 551 (21.9)	NA	
Semaglutide	2920 (4.12)	NA	
Liraglutide	1276 (1.80)	NA	
Insulin Glargine	NA	26 355 (63.4)	
Insulin Lispro	NA	12 636 (30.4)	
Insulin Aspart	NA	1068 (2.57)	
Insulin Degludec	NA	948 (2.28)	
Insulin Detemir	NA	570 (1.37)	

Source: US Food and Drug Administration Adverse Event Reporting System.

**Figure 1.**
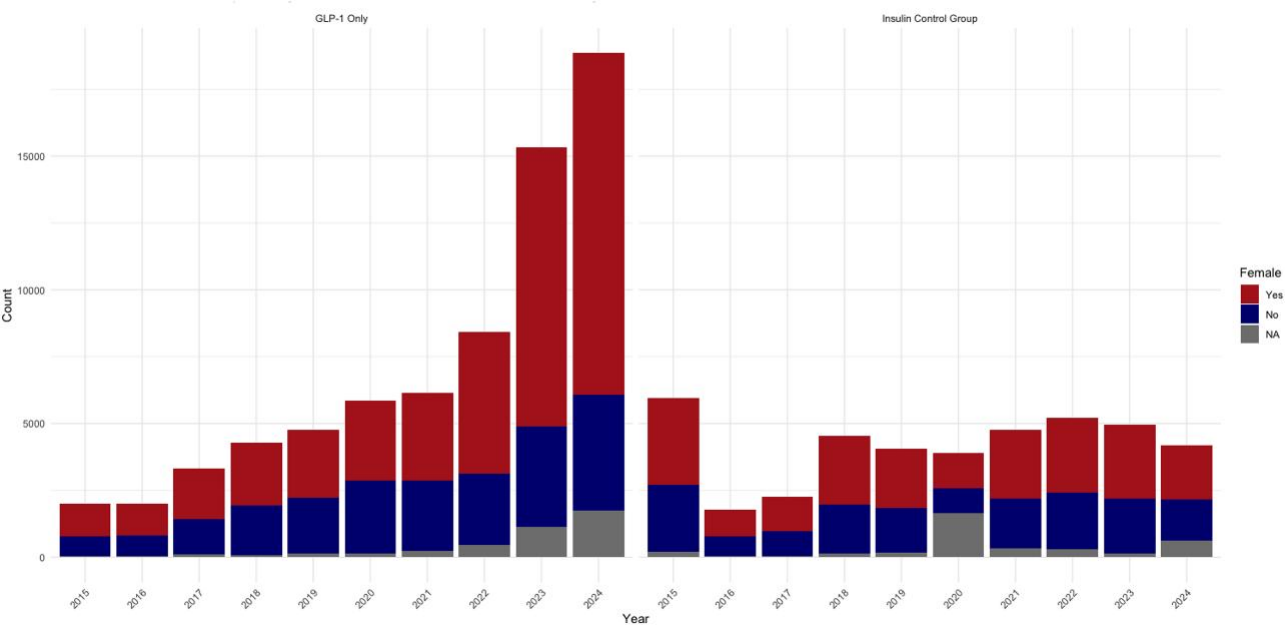
Count of adverse events reported by females for GLP-1s and Insulin from January 2015 to December 2024. Source: US Food and Drug Administration Adverse Event Reporting System.

**Figure 2.**
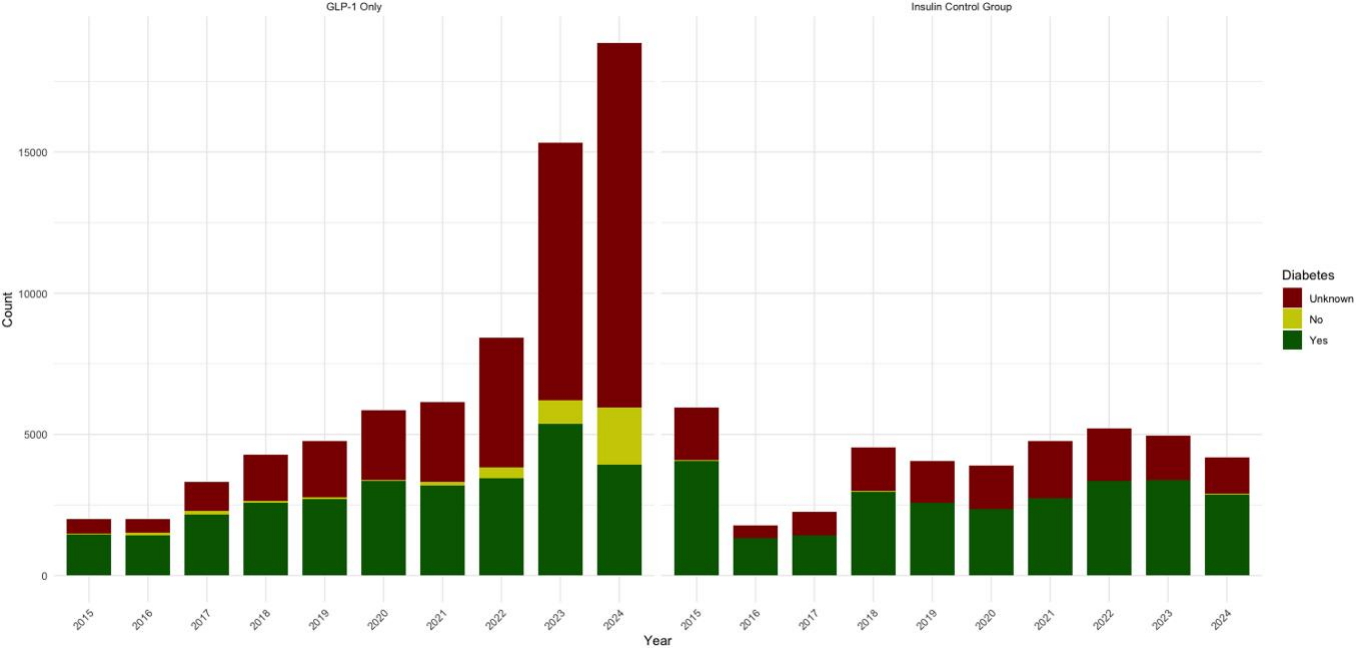
Count of included reactions for GLP-1s and insulin with a diabetes indication from January 2015 to December 2024. Source: US Food and Drug Administration Adverse Event Reporting System.

**Figure 3.**
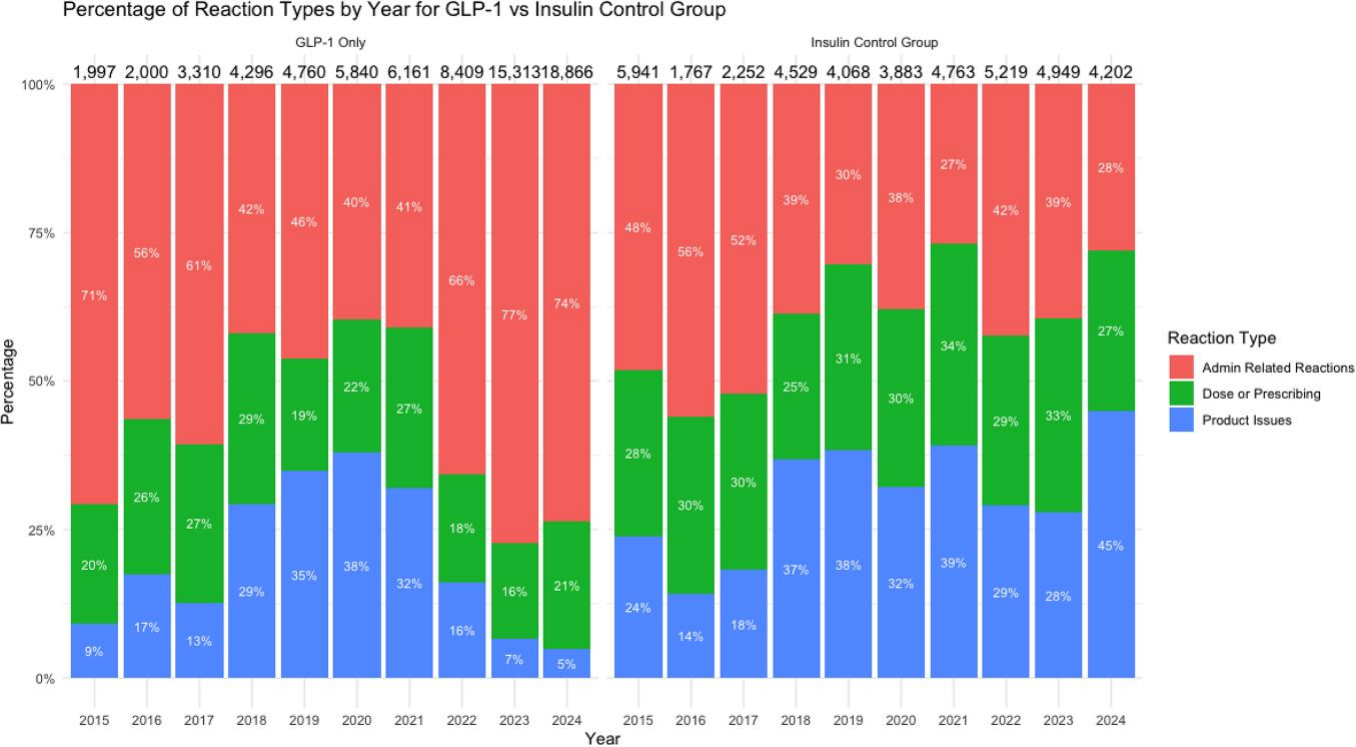
Distribution of product, dosing, or administration errors reported for GLP-1s and Insulin from January 2015 to December 2024. Source: US Food and Drug Administration Adverse Event Reporting System.

**Figure 4.**
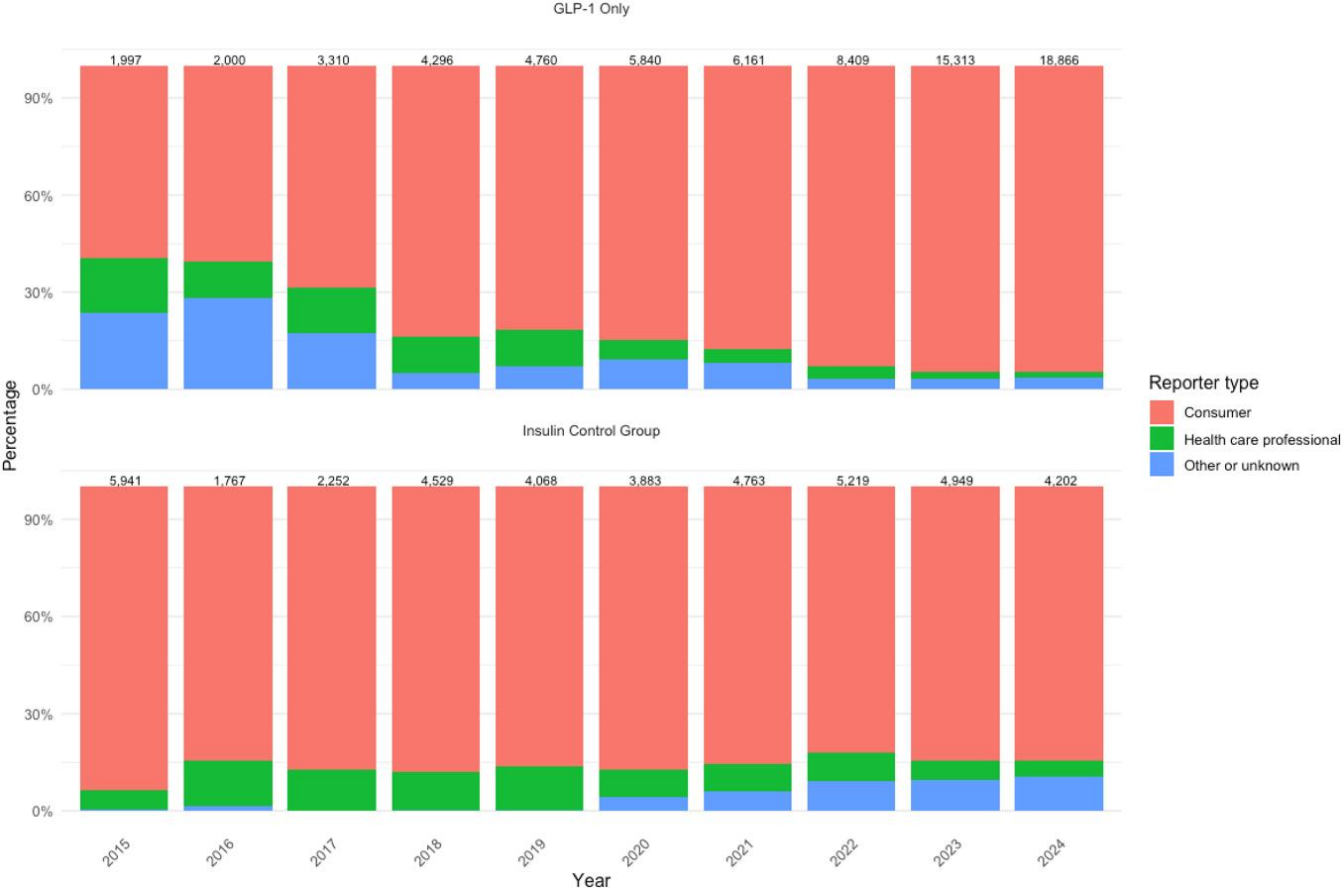
Distribution of reporter type for GLP-1 and insulin adverse event reports from January 2015 to December 2024.

Graphs depicting adverse event trends highlighted consistent patterns. The total number and proportion of females reporting adverse events for GLP-1s began rising steadily after 2021, with notable increases observed in 2022, 2023, and 2024 (Figure 1). Similarly, we observed a rise in the number and proportion of reports with an “Unknown” primary indication within the GLP-1 group starting in 2022 and continuing into 2023 and 2024 (Figure 2). Furthermore, administration-related reactions among GLP-1 reports increased sharply beginning in 2022, both in absolute counts and as a proportion of all reported events, with this shift becoming more pronounced in 2023 and 2024 (Figure 3 and eTable S3). In contrast, the distribution of administration, dosing, and product-related events among insulin reports remained relatively stable across the 2015-2024 period. Finally, across the study period, the majority of GLP-1 and insulin adverse event reports were submitted by consumers, with consumer reporting increasing over time for GLP-1s, while the proportion of health care professional reports remained relatively low and stable across both drug groups (Figure 4).

## Discussion

Our study assesses patterns in adverse events reported for two common classes of injectable drugs: GLP-1s and insulin. Consistently, the GLP-1 group showed a higher proportion of administration-related reactions compared to the insulin group, which is indicative of potential issues in drug delivery and handling. Although our analysis spans 2015-2024, the analytic focus is on changes occurring after the onset of national GLP-1 shortages in 2022. The extended pre-2022 period serves as a baseline to characterize reporting patterns prior to major shifts in demand, indication expansion, and supply disruption. In recent years, increasing utilization and demand for GLP-1s, especially for weight loss, has been well-documented.^17,18^ With this increase in utilization, we anticipated growth in total adverse events voluntarily reported to the FDA. The notable spikes in adverse events related administration, dosing, or product errors associated with the GLP-1s beginning in 2022 may be cause for concern.

The clinical consequences of dosing and administration errors differ fundamentally between insulin and GLP-1 receptor agonists. Insulin overdosing is typically associated with acute hypoglycemia, a well-recognized and immediately hazardous outcome that may arise from a single dosing error. In contrast, excessive dosing, rapid titration, or improper administration of GLP-1 therapies more commonly results in dose-dependent and cumulative toxicity rather than an isolated acute event. Reported adverse outcomes include gastrointestinal symptoms such as nausea, vomiting, and abdominal pain, which may progress to dehydration and electrolyte disturbances, as well as syncope, acute pancreatitis, and gallbladder-related complications.^4,19,20^ Importantly, these outcomes may arise from repeated overdosing, incorrect titration schedules, or deviations from recommended administration practices rather than from a single discrete error. Accordingly, administration- and dosing-related events represent clinically meaningful safety concerns for GLP-1 therapies and align directly with the medication-use problem categories examined in this analysis.

Notably, studies from US poison control centers, such as those by Gaw et al.,^21^ Marshall et al.,^22^ and Lambson et al.,^22^ support our findings as they are seeing an overall increase in the number of GLP-1 calls as well as calls related to improper administration, dosing errors, and patients reporting product malfunctions. Similar to our results, poison control centers are finding that GLP-1 adverse events are more frequently reported by females and the indication for the drugs are more frequently for weight loss or not provided during the call (higher rates of “unknown” indications in their data).^21,22^ Furthermore, published data shows GLP-1 prescriptions increased 6-fold from 2014 to 2022.^14^ However, we observed disproportionate changes in event types: administration and dosing-related reports increased from 19% to 27% of GLP-1 reports between 2021 and 2024, while product-related issues declined from 32% to 16%. Insulin reports remained stable throughout.

With the increased utilization and demand for GLP-1s, manufacturers struggled to maintain adequate supply which ultimately led to the FDA placing multiple GLP-1 products on the drug shortage list in the first quarter of 2022.^8^ Expanded reliance on nontraditional access pathways during the shortage period, including compounding pharmacies and online vendors, may plausibly affect how patients receive, prepare, and administer GLP-1 therapies, particularly if instructions, packaging, or patient education differ from those in traditional pharmacy settings. When a drug is placed on the drug shortage list, pharmacies are allowed to compound products that are “essentially copies” of the brand products using the same active pharmaceutical ingredients (APIs) purchased in bulk powder form from FDA-registered manufacturers.^23^ While the MedWatch adverse event forms used to submit reactions to FAERS have a “product type” flag that includes an optional check box for over-the-counter (OTC), compounded, generic, or biosimilar products,^24^ We found this field unsuitable for reliably identifying compounded products for several reasons. First, the field has high missingness rates (>85% of reports lack product type information). Second, we observed temporal instability in reporting patterns that appeared unrelated to actual changes in compounded product use. These issues likely reflect the voluntary and unstructured nature of reporting, variation in reporter understanding of product types, and lack of systematic verification of product source information. Additionally, there has been an increase in online companies potentially distributing GLP-1s illegally or distributing counterfeit products that we could not assess using FAERS data.^6^ While concerns over both compounded versions and counterfeit versions have been raised,^25^ we are unable to evaluate whether the administration, dosing, or product errors observed in our data are associated with these GLP-1 sources for patients. Also, we cannot determine from FAERS data whether individual adverse event reports involve FDA-approved products, compounded versions, or products obtained through other channels. While the temporal coincidence of increased administration and dosing error reports with the shortage period is suggestive, we cannot directly attribute these patterns to compounded products or nontraditional sources.

The absence of exposure denominators in FAERS limits the ability to estimate adverse event incidence or changes in per-patient risk over time. As a result, increases in the absolute number of reports associated with GLP-1 receptor agonists may reflect rapid growth in utilization rather than changes in underlying safety. In addition, FAERS does not reliably distinguish FDA-approved products from compounded, counterfeit, or otherwise nontraditional sources, precluding attribution of reported events to specific access channels. However, our analysis does not rely on report counts alone. Instead, we focus on changes in the distribution of reported event types within GLP-1 reports over time and contrast these patterns with a contemporaneous injectable comparison group (insulin). If increased utilization were the sole driver of observed trends, the relative proportions of administration-, dosing-, and product-related reports would be expected to remain stable. The marked post-2022 shift in administration- and dosing-related reports among GLP-1s—coupled with the absence of similar shifts among insulin reports—suggests a class-specific change in reported medication-use problems that warrants further investigation, particularly when considered alongside evidence from poison control center surveillance, even though true event rates cannot be determined.

## Conclusion

Our findings underscore the need for more patient education and measures to address adverse events associated with GLP-1 receptor agonists. Strengthening post-marketing surveillance is crucial to enhance the safe use of these prescription drugs.

## Supplementary Material

qxag023_Supplementary_Data

## References

[1] Han SH, Ockerman K, Furnas H, et al Practice patterns and perspectives of the off-label use of GLP-1 agonists for cosmetic weight loss. Aesthet Surg J. 2024;44(4):NP279–NP306. 10.1093/asj/sjad364 38085071

[2] Han SH, Safeek R, Ockerman K, et al Public interest in the off-label use of glucagon-like peptide 1 agonists (Ozempic) for cosmetic weight loss: a Google trends analysis. Aesthet Surg J. 2024;44(1):60–67. 10.1093/asj/sjad211 37402640

[3] Mahase E . GLP-1 shortages will not resolve this year, EMA warns, amid concern over off-label use. BMJ. 2024;385:q1448. 10.1136/bmj.q1448 38942431

[4] Long B, Pelletier J, Koyfman A, Bridwell RE. GLP-1 agonists: a review for emergency clinicians. Am J Emerg Med. 2024;78:89–94. 10.1016/j.ajem.2024.01.010 38241775

[5] Ockerman KM, Furnas HJ, Sheer A, Sorice-Virk S. Navigating the evolving roles of GLP-1 agonists safely and effectively. Aesthet Surg J. 2024;44(11):1241–1245. 10.1093/asj/sjae166 39041863

[6] Ashraf AR, Mackey TK, Schmidt J, et al Safety and risk assessment of no-prescription online semaglutide purchases. JAMA Netw Open. 2024;7(8):e2428280. 10.1001/jamanetworkopen.2024.28280 39093567 PMC11297364

[7] IQVIA Institute for Human Data Science . The use of medicines in the US 2024: usage and spending trends and outlook to 2028. 2024. Accessed September 28, 2025. https://www.iqvia.com/insights/the-iqvia-institute/reports-and-publications/reports/the-use-of-medicines-in-the-us-2024

[8] U.S. Food and Drug Administration . Drug shortages. Updated July 24, 2024. Accessed November 7, 2024. https://www.fda.gov/drugs/drug-safety-and-availability/drug-shortages

[9] U.S. Food and Drug Administration . Declaratory order: resolution of shortages of semaglutide injection products (Ozempic and Wegovy). US Food and Drug Administration. 2025. Accessed October 2, 2025 https://www.accessdata.fda.gov/scripts/drugshortages/

[10] U.S. Food and Drug Administration . FDA alerts health care providers, compounders and patients of dosing errors associated with compounded injectable semaglutide products. Updated July 26, 2024. Accessed July 26, 2024. https://www.fda.gov/drugs/human-drug-compounding/fda-alerts-health-care-providers-compounders-and-patients-dosing-errors-associated-compounded

[11] U.S. Food and Drug Administration . FDA's Adverse Event Reporting System (FAERS). Updated November 8, 2024. Accessed December 20, 2025. https://www.fda.gov/drugs/surveillance/fdas-adverse-event-reporting-system-faers

[12] Hoffman KB, Dimbil M, Kyle RF, et al A drug safety rating system based on postmarketing costs associated with adverse events and patient outcomes. J Manag Care Spec Pharm. 2015;21(12):1134–1143. 10.18553/jmcp.2015.21.12.1134 26679963 PMC10397967

[13] McCall KL, Mastro Dwyer KA, Casey RT, et al Safety analysis of compounded GLP-1 receptor agonists: a pharmacovigilance study using the FDA adverse event reporting system. Expert Opin Drug Saf. 2025;April 29:1–8. 10.1080/14740338.2025.2499670 40285721

[14] Park M, Carson AL, Conti RM. Linking medication errors to drug shortages: evidence from heparin supply chain disruptions caused by hurricane maria. Manuf Serv Oper Manag. 2025;27:1008–1024. 10.1287/msom.2023.0297

[15] U.S. Food and Drug Administration . FDA Adverse Event Reporting System (FAERS) quarterly data extract files. Updated July 30, 2024. Accessed September 1, 2024. https://fis.fda.gov/extensions/FPD-QDE-FAERS/FPD-QDE-FAERS.html

[16] von Elm E, Altman DG, Egger M, Pocock SJ, Gøtzsche PC, Vandenbroucke JP. The Strengthening the Reporting of Observational Studies in Epidemiology (STROBE) statement: guidelines for reporting observational studies. Ann Intern Med. 2007;147(8):573–577. 10.7326/0003-4819-147-8-200710160-00010 17938396

[17] Watanabe JH, Kwon J, Nan B, Reikes A. Trends in glucagon-like peptide 1 receptor agonist use, 2014 to 2022. J Am Pharm Assoc (2003). 2024;64(1):133–138. 10.1016/j.japh.2023.10.002 37821008

[18] Shi Q, Wang Y, Hao Q, et al Pharmacotherapy for adults with overweight and obesity: a systematic review and network meta-analysis of randomised controlled trials. Lancet. 2024;403(10434):e21–e31. 10.1016/S0140-6736(24)00351-9 38582569

[19] Wiener BG, Gnirke M, Vassallo S, Smith SW, Su MK. Challenges with glucagon-like peptide-1 (GLP-1) agonist initiation: a case series of semaglutide overdose administration errors. Clin Toxicol. 2024;62(2):131–133. 10.1080/15563650.2024.2322049 38470137

[20] Ismaiel A, Scarlata GGM, Boitos I, et al Gastrointestinal adverse events associated with GLP-1 RA in non-diabetic patients with overweight or obesity: a systematic review and network meta-analysis. Int J Obes. 2025;49(10):1946–1957. 10.1038/s41366-025-01859-6 PMC12532569 40804463

[21] Gaw CE, Hays HL, Kemp CA, et al Glucagon-like peptide-1 receptor agonist cases reported to United States Poison Centers, 2017-2022. J Med Toxicol. 2024;20(2):193–204. 10.1007/s13181-024-00999-x 38421490 PMC10959851

[22] Marshall S, Ryan E, Rivera J, Reynolds L, Atti S. GLP-1 receptor agonist exposures are increasingly common and generally associated with mild symptoms: a single poison center experience. J Med Toxicol. 2024;20(3):278–285. 10.1007/s13181-024-01008-x 38861153 PMC11288212

[23] U.S. Food and Drug Administration . Compounded drug products that are essentially copies of approved drug products under section 503B of the Federal Food, Drug, and Cosmetic Act: guidance for industry. Updated January 2018. Accessed October 2, 2025 https://www.regulations.gov/document/FDA-2016-D-1267-0032

[24] U.S. Food and Drug Administration . MedWatch forms for FDA safety reporting. Updated February 8, 2024. Accessed November 7, 2024. https://www.fda.gov/safety/medical-product-safety-information/medwatch-forms-fda-safety-reporting

[25] U.S. Food and Drug Administration . FDA's concerns with unapproved GLP-1 drugs used for weight loss. Updated October 2, 2024. Accessed November 7, 2024. https://www.fda.gov/drugs/postmarket-drug-safety-information-patients-and-providers/fdas-concerns-unapproved-glp-1-drugs-used-weight-loss

