# Supplementary material for: Adverse events administering glucagon-like peptide-1 receptor agonists: a cross-sectional study
Source: Health Aff Sch. 2026 Feb 3;4(2):qxag023. doi: 10.1093/haschl/qxag023 (PMC12927500; doi:10.1093/haschl/qxag023)
Supplement: qxag023_Supplementary_Data [file qxag023_supplementary_data.zip › APPENDIX.docx]

**APPENDIX**

**Adverse Events Reported for Prescription Weight Loss Drugs: A cross-sectional study of reports to the Food and Drug Administration Adverse Event Reporting System (FAERS)**

**eFigure 1.** Study flow diagram.

**eTable 1.** Active ingredients used to identify suspected product in adverse event report.

**eTable 2.** Categorization of reported reactions in FAERS.

**eTable 3**. Annual comparison of GLP-1 and insulin event reports related to product, dosing, or administration, 2015–2024.

**eFigure 1.**

**Caption:** Study flow diagram.


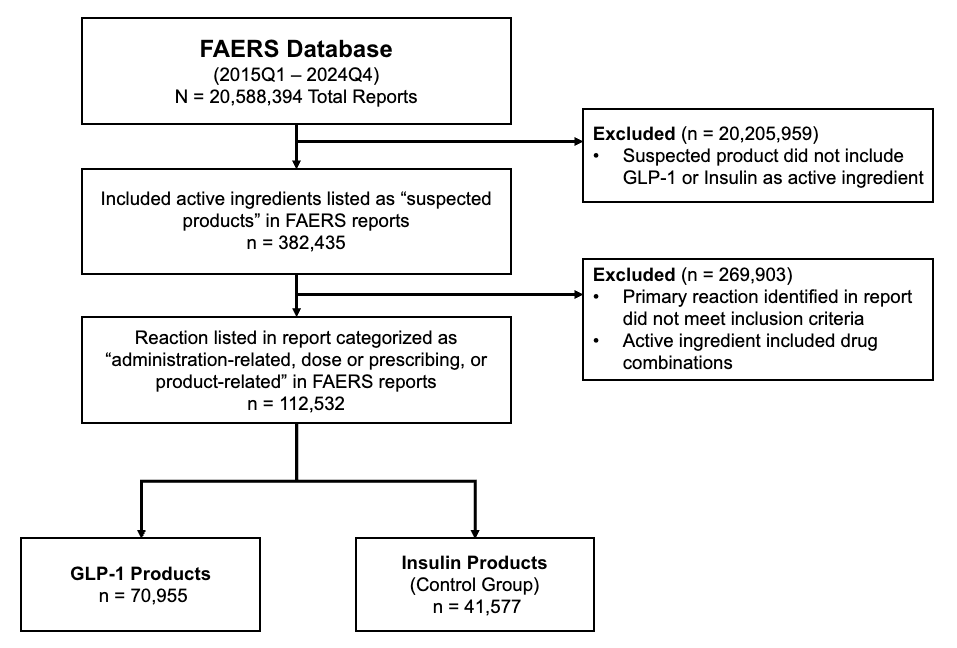


**Sources:** Food and Drug Administration Adverse Event Reporting System (FAERS).

**Notes:** Glucagon-like peptide-1 (GLP-1) receptor agonists and insulins identified in as a suspected product for a reported adverse reaction report were included.

**eTable 1.** Active ingredients used to identify suspected product in adverse event report.

| **Drug Category** | **Individual Drugs** |
| --- | --- |
| GLP-1 Only | "DULAGLUTIDE", "TIRZEPATIDE", "SEMAGLUTIDE", "EXENATIDE", "LIRAGLUTIDE" |
| Insulin Comparison group | "INSULIN ASPART", "INSULIN DEGLUDEC", "INSULIN DETEMIR", "INSULIN GLARGINE", "INSULIN LISPRO" |

**eTable 2.** Categorization of reported reactions in FAERS.

| **New Variable** | **Data in FAERS** |
| --- | --- |
| Administrative Related Reactions | "Injection site pain", "Injection site haemorrhage", "Injection site bruising", "Injection site mass", "Injection site pruritus", "Product dose omission issue", "Injection site erythema", "Wrong technique in device usage process", "Wrong technique in product usage process", "Product use issue", "Injection site injury", "Injection site extravasation", "Injection site vesicles", "Injection site irritation", "Incorrect route of product administration", "Injection site discolouration", "Injection site irritation", "Incorrect product administration duration", "Product administered at inappropriate site", "Needle issue", "Injection site nodule", "Injection site discomfort", "Injection site swelling", "Injection site urticaria", "Injection site rash", "Injection site hypersensitivity", "Product temperature excursion issue", "Intercepted product storage error", "Injection site discharge", "Injection site induration", "Injection site coldness", "Injection site indentation", "Injection site scar", "Incorrect product formulation administered", "Incorrect dosage administered", "Product administration error", "Product dose omission in error", "Injection site abscess", "Injection site infection", "Injection site haematoma", "Infusion site pain", "Injection site hypoaesthesia", "Drug administration error", "Incorrect drug administration duration", "Injection site cellulitis", "Administration site pain", "Incorrect route of drug administration", “Injection site laceration", "Application site pain", "Lack of injection site rotation", "Injection site reaction", "Product dose omission", "Intentional product use issue", "Wrong product administered", "Multiple use of single-use product", "Injection site inflammation", "Injection site paraesthesia", "Injection site warmth", "Wrong drug administered", "Incorrect dose administered", "Device use error", “Infusion site erythema”, “Localised infection”, “Infection”, “Bacterial infection”, “Fungal infection”, “Contusion”, “Injection site ulcer”, “Infusion site mass”, “Drug dispensed to wrong patient”, “Medication error” |
| Product Specific Issue | "Product tampering", "Poor quality product administered", "Product packaging issue", "Expired device used", "Counterfeit product administered", "Product physical issue", "Suspected counterfeit product", "Device occlusion"," Drug administered in wrong device", "Device ineffective", "Drug delivery system issue", "Incorrect dose administered by product", "Drug delivery system malfunction", "Product quality issue", "Expired product administered", "Product dispensing issue", "Device defective", "Device difficult to use", "Device use issue", "Device failure", "Device mechanical issue", "Device operational issue", "Device malfunction", "Device delivery system issue", "Injury associated with device", "Device leakage", "Device issue", "Product packaging quantity issue", "Product label confusion", "Device dispensing error", "Drug dispensing error", "Device breakage", "Product dispensing error",  "Drug dose omission by device", "Product substitution issue", "Incorrect dose administered by device", "Product storage error”, “Product preparation error”, “Product packaging difficult to open”, “Intercepted drug dispensing error”, “Drug eruption”, “Product communication issue”, “Product availability issue”, “Product use complaint”, “Liquid product physical issue”, “Intercepted product selection error”, “Product leakage”, “Intercepted product dispensing error”, “Product complaint”, “Intentional device misuse”, “Product odour abnormal”, “Product container issue”, “Product label issue”, “Circumstance or information capable of leading to medication error”, “Circumstance or information capable of leading to device use error”, “Complication associated with device”, “Product residue present”, “Product contamination”, “Product contamination physical”, "Product package associated injury", "Product solubility abnormal", “Suspected product quality issue”, "Product appearance confusion", "Product packaging confusion", "Product selection error", “Physical product label issue”, “Product design issue”, “Incorrect product storage”, “Poor quality drug administered” |
| Dosing or Prescribing Issues | "Wrong dose", "Overdose", "Underdose", "Product prescribing issue", "Drug prescribing error", “Product prescribing error”, “Drug titration error”, "Drug dose titration not performed", "Accidental underdose", "Accidental overdose", "Inappropriate schedule of product administration", "Drug dose omission", "Extra dose administered", "Intentional dose omission", “Inappropriate schedule of drug administration”, “Intentional overdose”, “Intentional product misuse”, “Intentional underdose” |

**eTable 3.**
**Caption:** Annual comparison of GLP-1 and insulin event reports related to product, dosing, or administration, 2015–2024.

|  | **GLP-1 Group** | | | **Insulin Group** | | |
| --- | --- | --- | --- | --- | --- | --- |
| **Year** | **Administration related** | **Dose or prescribing** | **Product Issues** | **Administration related** | **Dose or prescribing** | **Product Issues** |
| 2015 | 1,414  (71.0) | 401  (20.1) | 182  (9.1) | 2,860  (48.1) | 1,670  (28.1) | 1,411  (23.7) |
| 2016 | 1,129  (56.5) | 523  (26.2) | 348  (17.4) | 989  (56.0) | 527  (29.8) | 251  (14.2) |
| 2017 | 2,007  (60.6) | 886  (26.8) | 417  (12.6) | 1,176  (52.2) | 666  (29.6) | 410  (18.2) |
| 2018 | 1,804  (42.0) | 1,239  (28.8) | 1,253  (29.2) | 1,746  (38.6) | 1,119  (24.7) | 1,664  (36.8) |
| 2019 | 2,197  (46.2) | 905  (19.0) | 1,658  (34.8) | 1,235  (30.4) | 1,276  (31.4) | 1,557  (38.3) |
| 2020 | 2,316  (39.7) | 1,310  (22.4) | 2,214  (37.9) | 1,468  (37.8) | 1,168  (30.1) | 1,247  (32.1) |
| 2021 | 2,529  (41.0) | 1,664  (27.0) | 1,968  (32.0) | 1,280  (26.9) | 1,621  (34.0) | 1,862  (39.1) |
| 2022 | 5,523  (65.7) | 1,528  (18.2) | 1,358  (16.2) | 2,205  (42.2) | 1,502  (28.8) | 1,512  (29.0) |
| 2023 | 11,852  (77.4) | 2,448  (16.0) | 1,013  (6.6) | 1,950  (39.4) | 1,621  (32.8) | 1,378  (27.8) |
| 2024 | 13,902  (73.7) | 4,056  (21.5) | 908  (4.8) | 1,174  (28.0) | 1,138  (27.1) | 1,890  (45.0) |
